# Development and application of a wipe sampling method for detection of antibiotic surface contamination in hospital wards

**DOI:** 10.1093/annweh/wxaf067

**Published:** 2025-10-23

**Authors:** Carina A Nilsson, Elizabeth Huynh, Dallal Rashdan, Andreas Tinnert, Maria Hedmer, Monica Kåredal

**Affiliations:** Department of Occupational and Environmental Medicine, Skåne University Hospital, Lund SE-22381, Sweden; Department of Occupational and Environmental Medicine, Skåne University Hospital, Lund SE-22381, Sweden; Department of Occupational and Environmental Medicine, Skåne University Hospital, Lund SE-22381, Sweden; Department of Occupational and Environmental Medicine, Skåne University Hospital, Lund SE-22381, Sweden; Department of Occupational and Environmental Medicine, Skåne University Hospital, Lund SE-22381, Sweden; Division of Occupational and Environmental Medicine, Department of Laboratory Medicine, Lund University, Lund SE-22100, Sweden; Department of Occupational and Environmental Medicine, Skåne University Hospital, Lund SE-22381, Sweden; Division of Occupational and Environmental Medicine, Department of Laboratory Medicine, Lund University, Lund SE-22100, Sweden

**Keywords:** mass spectrometry, occupational exposure, wipe test

## Abstract

**Background:**

Antibiotics are handled in large amounts at hospitals at many different wards due to the wide range of bacterial infections that are treated. Unnecessary use and occupational exposure to antibiotics should be avoided due to the risk of bacterial resistance development and adverse health effects including skin and respiratory hypersensitivity reactions in persons handling these drugs.

**Objectives:**

To develop a wipe test method for sampling and quantification of surface contaminations of antibiotics, to assess the current contamination levels in Swedish hospitals, and to propose hygienic guidance values for antibiotics based on these measurements.

**Methods:**

A screening wipe test method and subsequent mass spectrometric analysis of 6 of the most frequently used antibiotics in healthcare was developed and applied in a screening campaign of 16 hospital wards. Wipe tests were sampled from surfaces such as workbenches, floors, storage shelves and handles in medicine rooms, patient rooms, rinsing rooms, utility rooms and corridors.

**Results:**

Antibiotics were detected in most of the samples (cefotaxime 84% positive samples, piperacillin 81%, cloxacillin 65%, metronidazole 53%, ciprofloxacin 20%, and penicillin V 14%). Median values ranged from not detected up to 160 pg/cm^2^ for the 6 different compounds and the highest results from an individual wipe sample were 27 × 10^6^ pg/cm^2^ (cefotaxime) and 3.0 × 10^6^ pg/cm^2^ (piperacillin). For cloxacillin, piperacillin, and metronidazole, lower levels of contamination were observed in medicine rooms when closed systems were used compared with samples collected in rooms where preparations were made without closed systems. Comparison of contamination levels showed that there were significant differences between different surface categories. Out of the most frequently detected antibiotics, ie cloxacillin, piperacillin, and cefotaxime, highest median values were found for surface categories floor and storage whereas lower median values were found for workbenches.

**Conclusion:**

A widespread environmental contamination of antibiotics was observed in hospital wards that potentially can contribute to the development of antibiotic-resistant bacteria as well as health impacts of exposed personnel. Probable sources include compounding, handling and administration of drug tablets, antibiotic contaminated waste as well as other sources such as excretions from patients and contaminated drug vials. Current surface cleaning routines do not sufficiently reduce spills and leakage regardless of source.

What's Important About This Paper? This study highlights that there is a widespread environmental contamination of antibiotics in hospitals wards in medicine rooms but also in common areas and patient rooms. The surface wipe method developed and applied in this study can be used to identify the sources of contamination so that levels can be reduced.

## Introduction

Antibiotics are common pharmaceuticals used for treatment and prevention of bacterial infections. Bacterial resistance against antibiotics is a growing global public health concern (WHO 2022). It was estimated that bacterial antimicrobial resistance (AMR) was linked to 1.27 million deaths in 2019 (Antimicrobial Resistance Collaborators 2022). There is a correlation between consumption and antibiotic resistance and the presence of antibiotics in the environment may contribute to the propagation (Reis et al. 2020; Sulis et al. 2022; Abejew et al. 2024). In one study personnel working in a pharmaceutical industry and exposed to high levels of penicillin dust showed higher prevalence of penicillin resistance than a control group suggesting that occupational exposure may also contribute to antibiotic AMR (Farshad et al. 2016).

In the US as well as in Europe, hazardous drugs, considered carcinogenic, mutagenic, or reprotoxic, have been identified and listed (NIOSH 2024; European Commission 2025). Most of the drugs on the list are antineoplastic drugs, and only a few antibiotics. According to EU directives and Swedish legislation, employers shall assess and manage identified occupational risks following the hierarchy of measures for risk prevention by elimination, substitution, engineering controls, administrative controls, and personal protective equipment, in that order (EU 1989; AFS 2023:10). As the use of antibiotics cannot be eliminated or substituted, implemented risks should be reduced by introducing engineering controls to isolate people from the hazard, including the use of biological safety cabinets (BSCs) and closed system drug-transfer devices (CSTD). However, compounding in safety cabinets or by applying CSTD is only required for cytotoxic (eg antineoplastic) drugs and not for antibiotics monitored in this study.

Antibiotics are handled in large amounts at hospitals at many different wards due to the wide range of infections that are treated. It is known that occupational exposure to antineoplastic drugs may occur in hospital wards and pharmacies during, eg, preparation, administration, during patient care or cleaning since residues of such drugs have been detected on surfaces in cabinets, computer equipment, waste bins, toilet seats, and floors (Connor et al. 2010; Labrèche et al. 2021; Leeman et al. 2025). Occupational exposure to antibiotics has been reported to cause non-allergic or allergic symptoms such as eye irritation, eczema, rhinitis, asthma, and anaphylaxis (Kim et al. 2011; Classen and Fuchs 2015; Whitaker 2016). The prevalence of drug hypersensitivity among healthcare personnel in Sweden is not known. However, sensitization to antibiotics via occupational exposure has been reported in several studies (Cetinkaya et al. 2007; Choi et al. 2010; Kim et al. 2012). Furthermore, reports show that exposure to antibiotics may cause allergic contact dermatitis (Pinheiro et al. 2018) or occupational asthma, rhinitis, and urticaria (Rudzki and Rebandel 1985; Moscato et al. 1995).

There is only a limited number of studies that have screened for how antibiotics are distributed in hospitals following spill and leakage of these drugs during handling (Nygren and Lindahl 2011a, 2011b; Sessink et al. 2019, 2024a). In 2008, Nygren and Lindahl (2011a, 2011b) developed a wipe test method and monitored surface levels of 12 antibiotics in Swedish hospital wards and they observed a widespread contamination. Strategies for reducing spills including cleaning routines were also suggested. In workplaces, implementing CSTD significantly reduced levels of antibiotic contaminations have been shown (Nygren and Lindahl 2011b; Sessink et al. 2019). However, no knowledge exists of current contamination surface levels in Swedish hospitals.

In Sweden, there is one occupational exposure limit (OEL) value for airborne penicillin dust of 0.1 mg/m^3^ (AFS 2023:14). For all other antibiotics, there are no OELs provided by the Swedish Work Environment Authority. Occupational exposure to antineoplastic drugs has been successfully monitored and managed by wipe sampling strategy in several countries including Canada, the Netherlands, Italy, Germany, and Austria showing reduced surface contamination levels (Crul and Simons-Sanders 2018; Dugheri et al. 2018; Chabut et al. 2022; Quartucci et al. 2023). In some countries, there are medical surveillance programs for personnel working with antineoplastic drugs and other hazardous drugs including cytotoxic compounds (Mathias et al. 2019). Hygienic guidance values (HGVs), performance-based values that are derived from measurements of existing levels found at workplaces fulfilling criteria of good hygiene practice, or threshold guidance values (TGVs) have been suggested for antineoplastic drugs as an attempt to reduce occupational exposure to protect the health of the workers when OELs are lacking (Hedmer and Wohlfart 2012; Kiffmeyer et al. 2013; Sottani et al. 2017).

The aim of the study was to develop a method for sampling and quantification of environmental surface contaminations of antibiotics most frequently used in Swedish hospitals. The aim was further to characterize the occupational exposure of antibiotics by surface monitoring of hospital wards in Sweden and to use the results from the measurements to propose HGVs for antibiotics.

## Methods

### Chemicals and materials

A selection of antibiotics was made based on prescription data from the south of Sweden reporting amounts used in the inpatient care of the public healthcare. The antibiotics included in the screening method were all top ranked substances based on amounts.

Cefotaxime sodium (CEFO, purity 97%, CAS-nr 64485-93-4), cefotaxime-d3 (purity 98%), ciprofloxacin-d8 (purity >95%, CAS-nr 1130050-35-9), cloxacillin-^13^C_4_, sodium salt (purity >95%), metronidazole (METRO, purity >95%, CAS-nr 443-48-1), metronidazole-d4 (purity >95%, CAS-nr 1261392-47-5), penicillin V potassium salt (PENV, purity >95%, CAS-nr 132-98-9), penicillin V-d5 (purity >95%), piperacillin-d5 (purity >95%) were purchased from Toronto research chemicals (Toronto, Canada). Ciprofloxacin (CIPRO, purity ≥98%, CAS-nr 85721-33-1), cloxacillin sodium salt monohydrate (CLOXA, purity ≥95%, CAS-nr 7081-44-9), piperacillin (PIPER, CAS-nr 66258-76-2), and formic acid (purity 97.5 to 98.5% for LC-MS) were purchased from Sigma-Aldrich (Saint Louis, MO, USA). Ethanol (analytical grade 95 to 96%) came from Solveco (Rosersberg, Sweden). Methanol (MeOH, LC-MS grade) was purchased from Merck KGaA (Darmstadt, Germany). Acetic acid (glacial, p.a. >99.8%) came from CCS Healthcare AB (Borlänge, Sweden). Non-woven wipes (5 × 5 cm, 45% polyester/55% cellulose) came from Dastex (Muggensturm, Germany).

The isotope-labelled antibiotics were used as internal standards (IS). A mixture of IS was prepared by dilution in ultrapure water at a final concentration of 0.5 µg/mL (IS solution). Ultrapure water was obtained from the water purifier system Milli-Q Integral 5 from Merck Millipore (Billerica, MA, USA).

### Wipe test sampling method

An in-house developed wipe sampling test was used with minor adjustments (Kåredal et al. 2022). In brief, a 400 cm^2^ surface, defined by a plastic frame (internal measures 20 × 20 cm), was wiped using 2 wipes, each moistened with 300 µL ultrapure water. The surface was wiped with a S-formed motion back and forth forming ∼3 to 4 S-formations covering the whole surface in one direction with one tissue and then with the second wipe in the other direction. Both wipes were placed in a 50 mL PP tube and stored at −20 °C until sample work-up. For each wipe sample, a new pair of protective gloves was used to avoid cross-contamination.

### Sample work-up procedure

To each sample, 100 µL IS solution and 10 mL ethanol was added. Samples were vortexed for 20 min at minimum speed using a Multi-Tube Vortexer (2500 Multi-Tube Vortexer, VWR International, Radnor, PA, USA), and 5 mL of the extract was transferred to 12 mL borosilicate glass tubes (Fisher Scientific, Hampton, NH, USA) and evaporated almost to dryness (miVac, Kovalent, Västra Frölunda, Sweden). Samples were dissolved in 150 µL 0.1% formic acid in methanol/ultrapure water mixture (15:85). Finally, samples were centrifuged at 1,000 × *g* for 5 min, and the solution was transferred to 1.5 mL glass vials with 200 µL inserts and centrifuged at 1,000 × *g* for 5 min before analysis.

A high and low quality control (QC) and standards were prepared by adding 100 µL of either quality control working solution or standard working solution to a 50 mL PP tube with 2 wipes, 600 µL ultrapure water and 100 µL IS solution and then treated according to the sample work-up procedure to generate QC_High_ 100 ng/sample, QC_Low_ 5 ng/sample, standard points 0.1; 1; 5; 50; 100; 200; and 1,000 ng/sample. A standard curve using weighted linear regression (1/x) was prepared. The calculated surface recovery was applied to compensate for losses during sampling.

### Chemical analysis

Samples were prepared and analyzed by liquid chromatography (LC, UFLCXR; Shimadzu Corporation, Kyoto, Japan) coupled to mass spectrometry (MS) on a QTRAP 4500 MS (Sciex, Framingham, MA, USA). An LC-MS/MS method based on multiple reaction monitoring (MRM) assays was developed for the simultaneous detection of 6 antibiotics and their corresponding isotope-labelled counterpart (Table S1).

Samples were injected (10 µL), and the compounds were separated on a reversed phase C18 column (Thermo Hypersil gold aqua C18, 3 µm, 50 × 2.1 mm, Thermo Fisher Scientific, Waltham, MA, USA) at a flowrate of 0.4 mL/min in a gradient elution (see Supplementary information) of mobile phase A (0.5% acetic acid) and B (0.5% acetic acid in methanol).

### Analytical validation

#### Detection limit, quantification limit, and reporting limit

The detection limit (LOD) and the limit of quantification (LOQ) of each compound was determined by calculating the concentration corresponding to 3 times and 10 times, respectively, of the standard deviation of the ratio between the peak area of the analyte and the peak area of the corresponding IS of a low calibrator (*n* = 10) prepared with wipe tissue and subjected to the sample work-up procedure.

#### Precision and accuracy

Within-day precision and accuracy were assessed by preparing and analyzing samples spiked with known amounts of all antibiotics at a high level (100 ng/sample, *n* = 10) and a low level (5 ng/sample, *n* = 10). The between-day precision was assessed by preparing and analyzing 2 samples spiked with known amounts of all antibiotics at a high level (100 ng/sample, *n* = 2) and a low level (5 ng/sample, *n* = 2) at 5 occasions over a period of 3 wks. Accuracy was determined by relating the quantified amount to the nominal amount. Acceptable level of precision was <30% (high level), <30% (low level), and acceptable level of accuracy was 70 to 130% (high and low level).

#### Surface recovery

A defined area of 400 cm^2^ was spiked with a mixed solution containing all drugs, 100 ng per compound. Wipe sampling and sample work-up and analysis were performed according to the protocol. Defined surfaces were composed of 3 different surface materials (*n* = 10 per surface material, 25 × 25 cm units cut inhouse); stainless steel, laminate (from a laminated shelf), and plastic (polyvinyl chloride flooring). Surface recovery was calculated as the percentage (ratio) between the concentration of spiked and wiped surface samples treated according to the sample work-up procedure and the concentration of a quality control sample (containing wipe tissue) spiked with the same amount of antibiotics (subjected to the sample work-up procedure).

#### Stability

The stability of each antibiotics in wipe samples stored at room temperature, at 4 °C, and at −20 °C was assessed by spiking wipe samples (2 tissues in 50-mL Falcon tubes, 100 ng/compound (100 µL of a mixture containing all antibiotics 1,000 ng/mL in ultrapure water)) and then determining the remaining level at time-points 0, 1, 2, 7, 14 days, 1, 2, and 6 mo (*n* = 5 per time-point and temperature).

### Safety routines

Preparation of standards, wipe sampling experiments with spiked surfaces, and sample preparations were all performed in a safety cabinet class II (BioClean, Suffolk, United Kingdom) and personal protection of nitrile gloves, safety goggles, and lab coat were used. All decontamination wipe material was discarded in a Pactosafe waste sealing container (Paxxo, Malmö, Sweden).

### Hospital screening campaign

#### Workplace measurements (study design)

In total, 16 different hospital wards, primarily specialized in medicine, surgery, orthopedics, infectious disease, and intensive care, all having an antibiotic-intense medical care and treatment, located at 5 different public hospitals were asked to participate in the antibiotics screening campaign and all accepted participation (Table S2). Measurements were carried out between December 2022 and June 2024.

All except one ward had medicine rooms for storage of pharmaceuticals (tablets, powder for i.v. injections or infusion, pre-prepared infusion bags, and syringes) in which preparations of solutions for infusions or injections of antibiotics also were done. Preparations were commonly made on workbenches with a disposal absorbent mat by connecting a CSTD, which should prevent the drug to be released outside the system, to the drug vial. Air vented dispensing units with an inbuilt particle filter (spike), which are open systems not preventing the release of vapors, were also provided at some wards to prevent aerosol formation during drug preparation and occasional wards only used this safety measure. The wards also received preprepared infusion bags and syringes of antibiotics. In summary, formulations of antibiotics including solutions, dry powder, and tablets were handled. A few wards had medicine rooms equipped with technical protection such as safety cabinets or forced ventilation but in the majority of wards such technical safety measures were lacking. Gloves (commonly nitrile) were used for personal protection and at some wards disposal aprons and face masks were used. Most wards had cleaning routines describing that workbenches should be cleaned with surface disinfectant (ethanol-based with surfactant). The frequency of cleaning varied.

#### Sampling points and survey questions

Wipe samples were collected in medicine rooms, rinsing rooms (eg used for cleaning and disinfection of urine bottles and waste bins), utility rooms, patient rooms, and corridors outside medicine rooms. Surfaces were categorized according to workbench (where compounding was performed; countertops, work area in safety cabinets or ventilated perfusion bench), storage (shelves and boxes), floors, handles (of storage drawers, refrigerators, doors and waste bins), equipment (such as drop counters, keyboards, monitors, surface of waste bin), drug vial and other surfaces.

The wards were asked to describe technical safety measures, frequency of antibiotic preparations, and cleaning routines (list of questions, Table S3).

#### Statistical evaluation

Statistical analysis was performed in IBM® SPSS® Statistics for Windows, version 30 (IBM Corp., Armonk, NY, USA). P-P plots showed that data was not normally distributed and were thus presented by median values and range (min, max-values). Kruskal–Wallis tests were used to compare data between groups (more than 2 independent groups) including surface concentrations between wards and between surface categories, respectively. Mann Whitney tests were used for comparisons between 2 groups including surface concentrations from wards applying technical measures and wards that did not and surface concentrations from 2 different surface categories, respectively. Values below the level of quantification were awarded a value of half the LOQ. Statistical significance was considered at *P* values below 0.05.

## Results

### Method validation

Matrix effects due to wipe material were evaluated (Table S4). The matrix effects that occurred due to the presence of wipe material were largely reduced by using internal standards for quantification. Thus, standards were prepared by including 2 wipe tissues to reflect the sample matrix.

The detection limits of the antibiotics were determined (Table 1). The slope of the standard curve was obtained from standards containing sample matrix, ie wipe material, with known amount of antibiotics added. Standard curves were linear in the range 0 to 1,000 ng substance/sample (R^2^ ≥ 0.99) for all antibiotics.

**Table 1.** Detection limits (LOD) and limit of quantification (LOQ) calculated for each antibiotic (n = 10 samples).

	CIPRO	CLOXA	PIPER	PENV	CEFO	METRO
LOD (ng/sample)	0.79	0.25	0.30	0.28	0.31	0.14
LOD (pg/cm^2^)	2.0	0.62	0.76	0.70	0.78	0.36
LOQ (ng/sample)	2.6	0.83	1.0	0.94	1.0	0.48
LOQ (pg/cm^2^)	6.6	2.1	2.5	2.3	2.6	1.2

A high within-day precision was obtained at the high level (QC_High_ 2.5% to 3.7%) for all antibiotics except CIPRO (18%), as well as at the low level (QC_Low_ 4.1% to 11%) for all antibiotics except CIPRO (22%; Table S5). The between-day precision was high ranging from 3.5% to 7.6% at the high level (QC_High_, except CIPRO 26%) and a variation between 3.7% and 8.9% at the low level (QC_Low_, CIPRO 21%; Table S6). The accuracy was found to be within 72% to 110% for all antibiotics and levels. Thus, the acceptance criteria were fulfilled for all antibiotics included in the screening method.

The recovery from spiked and wipe sampled surfaces varied between antibiotics in the screening method (Table S7). METRO showed high recoveries regardless of surface material, between 88% and 106%. CEFO showed a recovery above 80% when sampled from plastic and laminate surfaces but a lower recovery when sampled from a surface of stainless steel (64%). In general, lower recoveries of the compounds were observed at stainless steel. The lowest recovery was obtained for CIPRO sampled at stainless steel showing 16% recovery. Remaining recoveries were between 44% and 77%.

To assess the impact of storage conditions on the wipe samples, the recovery of spiked wipe samples was monitored over a period of up to 6 mo (Table S8). At room temperature, recovery was assessed up to 2 mo. No decomposition of CIPRO or METRO was observed during that time. Approximately 50% of CLOXA remained after 1 wk and <20% after 1 mo. For PIPER, PENV, and CEFO, ∼50% to 58% remained after 2 wks of storage in room temperature. The stability of refrigerated samples was assessed up to 6 mo of storage. No decomposition of CIPRO was detected after 6 mo, and the recovery of METRO was ∼90%. The recovery of CEFO was ∼90% after 2 wks. Of the remaining antibiotics (CLOXA, PIPER, and PENV), the recoveries were comparable with storage at room temperature up until 2 wks, after which no levels could be detected. Assessment of sampled stored at −20 °C showed that no decomposition of CIPRO and CEFO occurred up to 6 mo. Only a slight degradation of METRO was observed (95% recovery) after 6 mo. After 2 mo, 80% of CLOXA, 94% of PIPER, and 85% of PENV remained. In conclusion, samples should preferably be stored at −20 °C after sampling and analyzed within 2 mo of storage time.

### Measurements of surface contaminations in hospitals

**Fig. 1.**
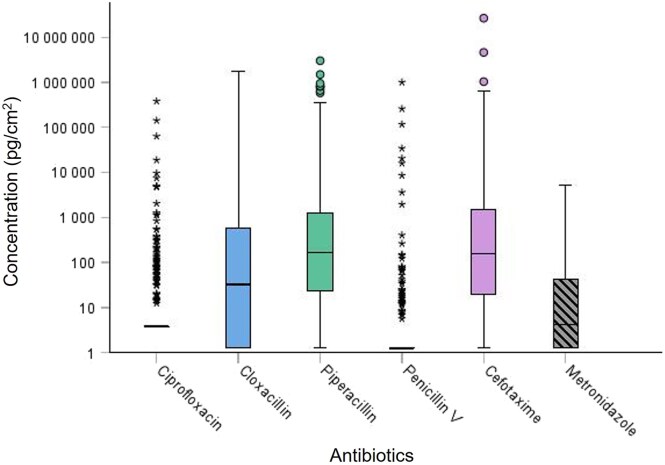
Boxplots demonstrating a summary of surface concentrations of antibiotics (logarithmic scale) from the whole data set (n = 313 wipe tests). The median value represented by the black horizontal line in the box. Outliers and extreme outliers represented by circles and stars, respectively.

In total, 13 wipe tests were sampled from 16 hospital wards and overall results showed that CEFO was most frequently detected (84% positive samples), followed by PIPER (81%), CLOXA (65%) and then METRO (53%), CIPRO (20%), and PENV (14%). Median values ranged from not detected (ND) up to 160 pg/cm^2^, and the highest results from an individual wipe sample were 27 × 10^6^ pg/cm^2^ (CEFO) and 3.0 × 10^6^ pg/cm^2^ (PIPER) (Fig. 1).

**Fig. 2.**
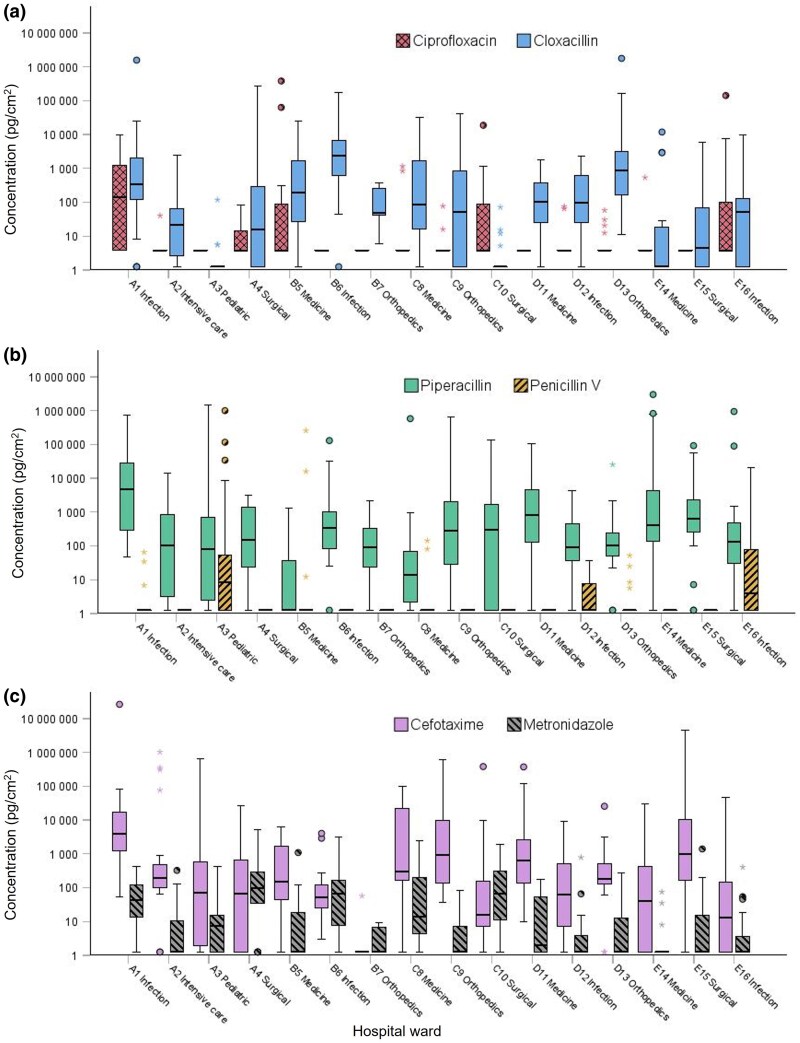
Boxplots demonstrating surface concentrations of antibiotics (logarithmic scale) measured in wipe samples from 16 hospital units (1 to 16) at 5 hospitals (A to E). Number of wipe samples collected from each unit: A1 n = 22, A2 n = 20, A3 n = 31, B4 n = 18, B5 n = 20, B6 n = 19, B7 n = 5, C8 n = 19, C9 n = 16, C10 n = 25, D11 n = 16, D12 n = 21, D13 n = 19, E14 n = 19, E15 n = 21, E16 n = 22. Wipe samples were collected from eg workbenches, floors, storage shelves, equipment and handles. Graphs representing a) ciprofloxacin (CIPRO) and cloxacillin (CLOXA), b) piperacillin (PIPER) and penicillin V (PENV), and c) cefotaxime (CEFO) and metronidazole (METRO).

Surface contamination of antibiotics from each ward was summarized (Fig. 2, Table S9). The median values for each ward ranged between ND and 4,900 pg/cm^2^ (PIPER). Surface levels of contamination observed between wards were significantly different (Kruskal–Wallis, *P* < 0.001).

A significant difference of surface levels of CLOXA, PIPER, and METRO was observed in samples collected from medicine rooms where CSTDs were used compared to samples collected in medicine rooms where preparations were made without CSTDs (Mann–Whitney *U* test, *P* < 0.05). Also, in wards where CSTDs were used, lower median values were recorded for the medicine rooms (Table 2).

**Table 2.** Environmental surface concentration of antibiotics in medicine rooms from wards that applied the use of CSTD and from wards that did not use CSTD, shown as median values.

	CIPRO	CLOXA	PIPER	PENV	CEFO	METRO
	Median (pg/cm^2^)					
Wards using CSTD^a^	ND^b^	49	190	ND	190	ND
Wards not using CSTD	ND	640	430	ND	270	19

^a^CSTD, closed system drug-transfer devices.

^b^ND, not determined, value below the limit of quantification.

Surface concentrations were summarized according to category of surface and to subcategories (sampling locations, Table 3). Comparison of contamination levels showed that there were significant differences between surface categories for all antibiotics (Kruskal–Wallis, *P* < 0.05). For CIPRO, PIPER, and METRO, significance level (*P*) was <0.001.

**Table 3.** Environmental surface concentration of antibiotics based on surface category shown as median, ranges, and the percentage of positive samples (ie detected above the LOQ).

Surface category	n^a^	CIPRO		CLOXA		PIPER		PENV		CEFO		METRO	
		Median (min-max) (pg/cm^2^)	>LOQ^b^ (%)	Median (min-max) (pg/cm^2^)	>LOQ (%)	Median (min-max) (pg/cm^2^)	>LOQ (%)	Median (min-max) (pg/cm^2^)	>LOQ (%)	Median (min-max) (pg/cm^2^)	>LOQ (%)	Median (min-max) (pg/cm^2^)	>LOQ (%)
***Workbench all surfaces***	64	ND^c^ (ND-7,500)	17	35 (ND-1.6 × 10^6^)	78	190 (ND-3.0 × 10^6^)	84	ND (ND-1.0 × 10^6^)	22	100 (ND-2.7 × 10^7^)	94	3.7 (ND-510)	53
Medicine room	56	ND (ND-7,500)	20	37 (ND-1.6 × 10^6^)	80	330 (ND-3.0 × 10^6^)	89	ND (ND-1.0 × 10^6^)	25	150 (ND-2.7 × 10^7^)	96	5.9 (ND-510)	59
Rinsing room	6	ND (ND-ND)	0	5.4 (ND-2,500)	50	6.1 (ND-9.8)	67	ND (ND-ND)	0	34 (ND-100)	67	ND (ND-5.4)	17
Medicine trolley	1	ND	0	50	100	ND	0	ND	0	73	100	ND	0
Work table operation	1	ND	0	5.9	100	330	100	ND	0	ND	0	ND	0
***Storage all surfaces***	51	ND (ND-3.8 × 10^5^)	43	70 (ND-6,000)	69	340 (ND-1.5 × 10^6^)	80	ND (ND-2.6 × 10^5^)	22	330 (ND-79,000)	92	ND (ND-1,400)	39
Storage shelf/box in medicine room	49	ND (ND-1,200)	41	70 (ND-6,000)	69	350 (ND-1.5 × 10^6^)	84	ND (ND-140)	18	450 (ND-79,000)	94	ND (ND-1,400)	41
Medicine box trolley in corridor	2	2.2 × 10^5^ (6.3 × 10^4^ to 3.8 × 10^5^)	100	14 (ND-28)	50	ND (ND-ND)	0	1.4 × 10^5^ (1.6 × 10^4^ to 2.6 × 10^5^)	100	34 (ND-67)	50	ND (ND-ND)	0
***Floor all surfaces***	82	ND (ND-1.4 × 10^5^)	29	90 (ND-1.8 × 10^5^)	77	450 (ND-9.4 × 10^5^)	96	ND (ND-3,600)	17	210 (ND-1.0 × 10^6^)	98	64 (ND-2,600)	91
Medicine room in front of workbench	27	ND (ND-1.4 × 10^5^)	37	180 (ND-1.8 × 10^5^)	81	1,200 (11 to 9.4 × 10^5^)	100	ND (ND-3,600)	22	720 (27 to 1.0 × 10^6^)	100	110 (ND-2,600)	96
In front of waste bin medicine room	14	ND (ND-5,000)	36	1,900 (ND-4.1 × 10^4^)	93	470 (52 to 2.8 × 10^4^)	100	ND (ND-52)	14	530 (20 to 3.7 × 10^5^)	100	170 (5,1 to 1,300)	100
Corridor	16	ND (ND-560)	19	14 (ND-1,100)	69	100 (ND-1,300)	88	ND (ND-25)	13	70 (3.0 to 1.2 × 10^4^)	100	19 (ND-420)	88
Patient room	4	ND (ND-15)	25	12 (ND-27)	50	6,100 (200 to 1.4 × 10^4^)	100	ND (ND-8.5)	25	160 (14 to 220)	100	320 (130 to 430)	100
Patient room WC	6	ND (ND-150)	33	ND (ND-240)	33	1,900 (ND-3.5 × 10^4^)	83	ND (ND-8.2)	17	320 (3.1 to 6,700)	100	30 (ND-110)	67
Rinsing room	13	ND (ND-69)	23	33 (ND-1.1 × 10^4^)	85	430 (61 to 7,800)	100	ND (ND-13)	15	200 (8.7 to 1,000)	100	20 (ND-2,500)	85
Operating room	2	ND (ND-ND)	0	120 (48 to 250)	100	57 (23 to 91)	100	ND (ND-ND)	0	ND (ND-ND)	0	8.0 (6.6 to 9.4)	100
Handles all surfaces	75	ND (ND-1,100)	4	ND (ND-2.8 × 10^5^)	45	36 (ND-9.0 × 10^4^)	57	ND (ND-8,600)	4	130 (ND-4.6 × 10^6^)	65	ND (ND-5,200)	21
Door handle medicine room	14	ND (ND-ND)	0	ND (ND-1,400)	29	4.3 (ND-430)	50	ND (ND-18)	7	150 (ND-3.0 × 10^4^)	71	ND (ND-16)	14
Door handle rinsing room	6	ND (ND-ND)	0	ND (ND-150)	17	ND (ND-620)	33	ND (ND-ND)	0	ND (ND-1,200)	33	ND (ND-ND)	0
Door handle patient room	3	ND (ND-390)	33	ND (ND-13)	33	ND (ND-51)	33	ND (ND-ND)	0	14 (ND-41)	67	ND (ND-ND)	0
Refrigerator handle medicine room	11	ND (ND-ND)	0	ND (ND-600)	27	50 (ND-1,200)	64	ND (ND-8,600)	9	100 (ND-8,700)	73	ND (ND-78)	36
Lid/handle waste bin	23	ND (ND-ND)	0	ND (ND-8,700)	43	36 (ND-2,800)	52	ND (ND-12)	4	70 (ND-4.6 × 10^6^)	57	ND (ND-110)	9
Ledge of storage box consumables	12	ND (ND-1,100)	17	100 (ND-5,700)	75	730 (ND-9.0 × 10^4^)	92	ND (ND-ND)	0	830 (ND-3.8 × 10^5^)	83	3.9 (ND-5,200)	50
Ledge/handle of storage box antibiotics	2	ND (ND-ND)	0	1.4 × 10^5^ (1.2 × 10^4^ to 2.8 × 10^5^)	100	310 (ND-630)	50	ND (ND-ND)	0	760 (ND-1,500)	50	63 (35 to 92)	100
Water tap	4	ND (ND-ND)	0	640 (300 to 1,800)	100	56 (ND-3.6 × 10^4^)	50	ND (ND-ND)	0	810 (ND-3,400)	75	ND (ND-ND)	0
***Equipment all surfaces***	29	ND (ND-ND)	0	25 (ND-4.4 × 10^4^)	55	91 (ND-6.6 × 10^5^)	83	ND (ND-70)	3	70 (ND-5.9 × 10^5^)	83	11 (ND-3,100)	55
Control panel infusion pump	13	ND (ND-ND)	0	ND (ND-180)	23	31 (ND-6.6 × 10^5^)	85	ND (ND-ND)	0	55 (ND-5.9 × 10^5^)	69	38 (ND-780)	62
Computer equipment medicine room	10	ND (ND-ND)	0	3,700 (ND-2.5 × 10^4^)	80	250 (ND-3.2 × 10^4^)	80	ND (ND-70)	10	190 (3.0 to 2,900)	100	7.8 (ND-3,100)	60
Waste bin medicine room	3	ND (ND-ND)	0	870 (390 to 4.4 × 10^4^)	100	130 (39 to 1,300)	100	ND (ND-ND)	0	3,100 (42 to 7,800)	100	67 (ND-280)	67
Scale rinsing room	1	ND	0	ND	0	ND	0	ND	0	ND	0	ND	0
Equipment surgery	2	ND (ND-ND)	0	210 (42 to 380)	100	1,100 (ND-2,200)	50	ND (ND-ND)	0	29 (ND-57)	50	ND (ND-ND)	0
***Drug vial with CLOXA***	1	ND	0	1.8 × 10^6^	100	2.5 × 10^4^	100	ND	0	ND	0	ND	0
***Other surfaces, all***	11	ND (ND-1.9 × 10^4^)	18	ND (ND-4,000)	36	140 (ND-1.3 × 10^5^)	91	ND (ND-120)	18	19 (ND-2.4 × 10^5^)	55	ND (ND-350)	45
Underneath ventilated workbench medicine room	1	1.9 × 10^4^	100	ND	0	1.3 × 10^5^	100	ND	0	9,900	100	350	0
Door opener outwards	6	ND (ND-ND)	0	ND (ND-520)	17	140 (79 to 4,300)	100	ND (ND-120)	17	ND (ND-440)	33	ND (ND-18)	33
Bedtable patient room	1	ND	0	ND	0	ND	0	ND	0	ND	0	ND	0
Laminated document/binder medicine room	3	ND (ND-32)	33	650 (2.0 to 4,000)	100	910 (190 to 5.8 × 10^4^)	100	ND (ND-25)	33	120 (19 to 2.4 × 10^5^)	100	4.7 (ND-18)	67

^a^Number of wipe tests analyzed.

^b^Percentage of samples above the limit of quantification (LOQ).

^c^ND, not determined, value below the limit of quantification.

Out of the most frequently detected antibiotics, ie CLOXA, PIPER, and CEFO, highest median values were found for surface categories floor and storage whereas lower median values were found for workbenches. The highest frequency of positive samples was found in the category floor for all antibiotics (CIPRO 29%, PIPER 96%, CEFO 98%, METRO 91%) except for PENV (22% work area, 17% floor) and CLOXA (78% work area, 77% floor). Within the category floor, the highest amounts of antibiotics were measured in wipe tests from floors in front of workbenches. In wipe tests sampled from medicine rooms, there was a significant difference (Mann–Whitney *U* test, *P* < 0.05) between levels found at floors and levels found at work areas for all antibiotics except CIPRO (*P* = 0.056) and PENV (*P* = 0.41). The median values of antibiotics were also consequently higher in wipe tests sampled from floors compared with work areas for those antibiotics having significant differences (Table 4).

**Table 4.** Environmental surface levels of antibiotics in medicine rooms presented in surface category workbench and floor, respectively, shown as median values.

	CIPRO	CLOXA	PIPER	PENV	CEFO	METRO
Median value workbench medicine room (pg/cm^2^)	ND^a^	37	330	ND	150	5.9
Median value floor medicine room (pg/cm^2^)	ND	500	980	ND	720	120

^a^ND, not determined, value below the limit of quantification.

In wipe samples collected from storage shelves and boxes for antibiotics in medicine rooms, contamination of antibiotics were observed to different extent depending on the compound (18 to 94% positive samples), with a highest value of 1.5 × 10^6^ pg/cm^2^ (PIPER).

Generally, lower median levels were observed on handles, but the distribution of contamination was quite broad within this category. The highest proportion of positive wipe tests were seen for handles and edge strips of boxes and drawers where consumables such as adaptors for spike and CSTDs were kept (CLOXA 75% positive samples, PIPER 92% och CEFO 83%). Highest levels of contamination of antibiotics within the category handles was found at handles of waste bins (CEFO 4.6 × 10^6^ pg/cm^2^) and on handles of boxes storing pharmaceuticals (CLOXA 2.8 × 10^5^ pg/cm^2^). On in- and outward door handles to rinsing and patient rooms, there was a low proportion of positive wipe tests and in general low levels of contamination whereas in- and outward door handles to medicine rooms showed slightly higher levels of contamination for some compounds (highest CEFO 3.0 × 10^4^ pg/cm^2^).

A high proportion of positive wipe tests were found when sampling equipment including computer equipment in medicine rooms (keyboards, screen, and computer mouse with highest value of 3.2 × 10^4^ pg/cm^2^ for PIPER), surfaces of waste bins (highest individual value of 4.4 × 10^4^ pg/cm^2^ for CLOXA) and control panels of infusion pumps (highest individual value of PIPER 6.6 × 10^5^ pg/cm^2^).

One drug vial from a drug packaging was wipe sampled and external contamination of 2 antibiotics were detected on the surface, 1.8 × 10^6^ pg/cm^2^ of CLOXA and 2.5 × 10^4^ pg/cm^2^ of PIPER.

### Hygienic guidance values

Based on data from the 16 wards where the antibiotics were handled, the 75th and 90th percentiles were determined (Table 5).

**Table 5.** Substance-specific hygienic guidance values based on the 90th and 75th percentile, respectively, of the surface level of antibiotics measured at 16 hospital wards located at 5 public Swedish hospitals where antibiotics was handled.

Compound	CIPRO	CLOXA	PIPER	PENV	CEFO	METRO
90th percentile (pg/cm^2^)	130	3,600	9,600	20	2.2 × 10^4^	260
75th percentile (pg/cm^2^)	ND^a^	590	1,300	ND	1,600	45

^a^ND, not determined, value below LOQ.

## Discussion

### Sampling and analysis method

The developed wipe test method is applicable for monitoring of antibiotic surface contamination in hospitals as it can simultaneously screen for 6 of the currently most frequently used antibiotics. Compared to previously described wipe test methods for quantification of antibiotics on surfaces, the detection levels reported for our method were in the same order or lower than previously reported (Nygren and Lindahl 2011a; Sessink et al. 2019, 2024a). Overlapping antibiotics in all methods were CIPRO, METRO (Nygren and Lindahl 2011a), and PIPER and CEFO (Sessink et al. 2019, 2024a). Thus, the current wipe test method is highly relevant for the measurement of current environment surface contamination of antibiotics.

Sampling procedure as well as the analytical method were validated and met the predetermined acceptance criteria regarding precision and accuracy. Matrix effects were observed, but these were shown to be sufficiently managed by the implementation of internal standards. The surface recovery was also assessed as it affects the yield of each antibiotic that is sampled from the surface and the recovery was applied when assessing surface contamination levels to adjust for expected losses. The recoveries for the screened antibiotics varied depending on compound but were in general higher for surfaces of laminate and plastic and lowest on stainless steel. The low recovery of CIPRO from stainless steel (16%) could potentially be linked to the absorption capacity of CIPRO to polyvalent metal ions (Imaoka et al. 2014). All other recoveries were between 37% and 106%.

### Screening campaign in hospitals

The method was applied in a hospital survey where surface contamination of antibiotics was screened for at 16 wards situated at 5 different hospitals in southern Sweden. Overall results from total 313 wipe tests showed a widespread surface contamination of several of the measured antibiotics. The most frequently detected antibiotics was CEFO, positive in 84% of all samples, and the least detected was PENV, positive in 14% of samples. Levels ranged between not detected up to 27 × 10^6^ pg/cm^2^. There is one earlier comprehensive study carried out in 2008 that measured contamination of antibiotics in 21 hospital wards in Sweden (Nygren and Lindahl 2011b) and 2 smaller studies that carried out repeated measurements of antibiotics contaminations of European hospital wards (Sessink et al. 2019, 2024a). Two of the antibiotics measured by Nygren and Lindahl, CIPRO och METRO, showed higher median values (48 and 61 pg/cm^2^, Nygren and Lindahl 2011b), compared to our study (CIPRO ND and METRO 4.3 pg/cm^2^). However, results for other antibiotics, eg PIPER and CEFO, which were not included in the screening method of the previous study, showed much higher median values in our study (PIPER 160 pg/cm^2^ and CEFO 150 pg/cm^2^) likely a consequence of being used in higher amounts. In the pilot study by Sessink et al., monitoring surface levels of one Swedish hospital observed substantially higher median values measured when not using CSTD (PIPER 3.6 × 10^4^ and CEFO 9,000 pg/cm^2^, Sessink et al. 2019). There was however a large distribution of levels between the different wards in our study with median values ranging between levels below the limit of quantification up to 4,900 pg/cm^2^ (PIPER). There are several factors likely affecting the differences in contamination levels of antibiotics at the different wards, eg which technical control measures that were applied during preparations, other work measures, cleaning routines, and amount of antibiotics handled at each ward. In European hospital wards, comparable levels of antibiotics were observed at surfaces such as workbenches for compounding and floors close to waste bins where drug vials were discarded (Sessink et al. 2024a).

In our study, surface contaminations of antibiotics were observed in safety cabinets and workbenches where preparations of infusions were performed but also on secondary surfaces such as floors, equipment, and handles. Antibiotics was also detected in wipe samples from storage shelves and boxes and on the surface of a drug vial. For workplaces to reduce levels of pharmaceutical contamination in a structured manner, it is important to know the point source of contamination. Based on the result of earlier and the current study, likely sources of contamination derive from compounding, antibiotics contaminated waste from compounding or patient care, administration of antibiotics (tablets, oral and infusion), patient care (excretions) but also likely from surface contaminated primary drug vials as indicated by the results of this study (Nygren and Lindahl 2011b; Sessink et al. 2019). It is known from hazardous drug surveillance, eg surface monitoring of antineoplastic drugs studied in numerous papers, that spills can occur during compounding or administration and that workbenches and floors in front of BSCs are frequently contaminated surfaces (Delafoy et al. 2023). Also, traces of antineoplastic drugs are commonly found on storage shelves, infusion pumps, computer equipment, infusion bags, and on floors in patient lavatories among other surfaces when hospital environments were monitored by wipe sampling (Hedmer et al. 2008; Ndaw and Remy 2023; Leeman et al. 2025). Surveys of surface contaminations from antineoplastic drugs in healthcare and pharmacies presenting the 90th percentile of 346 pg/cm^2^ in pharmacy areas and 443 pg/cm^2^ in patient care units (Sottani et al. 2022), 117 pg/cm^2^ (Kiffmeyer et al. 2013), 58 pg/cm^2^ (Dugheri et al. 2018), 979 pg/cm^2^ storage shelves and boxes (Schierl et al. 2009). In comparison, the 90th percentile of surface levels for cefotaxime observed in our study was 22,000 pg/cm^2^ indicating that surface residues of antibiotics are substantially higher than surface contaminations of antineoplastic drugs.

A significant difference between levels of antibiotics found on surfaces in medicine rooms of wards using CSTD compared to wards not using CSTD was observed, and the median values were consistently lower for these antibiotics at workplaces using CSTD. This is also supported by other studies where it was shown that the use of closed systems during compounding significantly decreased spills and leakage of pharmaceuticals (Nygren and Lindahl 2011b; Vyas et al. 2016; Bartel et al. 2018; Sessink et al. 2019, 2024b; Piccardo et al. 2021). Even though most wards used closed systems for compounding, antibiotics were frequently detected in medicine rooms and in levels as high as 4.6 µg/cm^2^ (ie 4.6 × 10^6^ pg/cm^2^). It is however known that closed systems not completely limit spills and leakages. An evaluation of 6 commercially available CSTDs aiming to control how well vapors were contained by the devices concluded that only 3 of the devices met the criteria set for the NIOSH test protocol, releasing vapor below the quantifiable threshold (Halloush et al. 2020). In our study, only a few wards stated that CSTDs were the only technical measure used, implying that most of the wards also permitted the use of systems other than closed systems. Air-vented dispensing units with an inbuilt particle filter (spike) is an open system that can release any vapors formed during compounding into surrounding air. Consequently, contamination may be a result of such use.

Sources of antibiotic contamination other than compounding include handling of tablets when these for example are split into smaller pieces. The outer surface of primary drug vials is also a possible source for spread of antibiotics as shown by the positive wipe sample collected from a CLOXA drug vial in this study. Contamination of such primary packages may contribute to contamination in wards as it is an unknown source for personnel handling them. Moreover, a source of antibiotics contamination may very well be related to the treatment of patients as some compounds are excreted to a large extend in an unmetabolized form, eg up to 60% of CEFO will be excreted in urine (Nielsen et al. 1980). Antibiotic residues are also affected by cleaning routines (cleaning method and frequency). At a few wards, levels of antibiotics were shown for compounds not specified to be used at the ward. Similar results were also observed by Nygren and Lindahl (2011b). It could be resulting from that the compound was indeed used although the staff was unaware of this, or from earlier use and that levels from that use remained, or possibly that the contamination come from patient excretions treated with antibiotics and moved from another ward. In this study, cleaning routines varied between wards as some wards had instruction of cleaning before and after each preparation whereas other cleaned more rarely. That high levels of antibiotics were detected on floors and other secondary surfaces suggests that regardless of source the actual spill and leakage was not reduced completely by the current cleaning routines. The finding also indicates that more knowledge is needed to introduce cleaning routines that efficiently reduces the levels of antibiotics when cleaning. This was shown in 2 previous studies that included an intervention step in their studies by which a follow-up measurement was done after giving suggestion for improvement of the cleaning method (more frequent and optimized cleaning routines). In several of the assessed effects, the levels of contamination were in fact not reduced at all, and in some cases, more compounds could be detected after cleaning than before (Nygren and Lindahl 2011b; Sessink et al. 2024a). Sessink et al. (2024a) concluded that implementation of a CSTD would be a more effective measure to reduce contamination levels. Further studies are required to identify and conclude the contributions of different sources to the overall environmental contamination of antibiotics. The presence of antibiotics contaminated surfaces in healthcare may increase the risk of occupational exposure leading to sensitization and hypersensitivity reactions in exposed personnel (Whitaker 2016).

Current European as well as Swedish legislation establish that employers should take action to prevent exposure by applying hierarchy of controls eg minimizing the exposure by for example using closed systems or BSCs (AFS 2023:10). There are no specific guidelines for antibiotics since most of these are not identified as carcinogenic, mutagenic, or reprotoxic and thus probably identified as less harmful. However, some antibiotics have inherent sensitizing properties and for such chemicals usually a low amount is sufficient to cause adverse effects. To reduce or eliminate this risk, the hierarchy of controls should be applied by eg using BSCs and CSTD. Exposure to antibiotics via inhalation have been reported as healthcare workers have stated that they could smell the drugs indicating that vapors or emissions have reached the air from spills or leakages (Sessink et al. 2019). There is one OEL in air for penicillin but for other antibiotics there are no OEL values. Hygienic guidance values have been suggested for antineoplastic drugs as a tool to decrease the occupational exposure, but there are currently no HGVs suggested for antibiotics (Hedmer and Wohlfart 2012; Kiffmeyer et al. 2013; Sottani et al. 2017). HGVs are not health based but instead practical, achievable levels, based on the 90th percentile of measurements from work settings where the pharmaceuticals are handled. We propose hygienic guidance values for 6 antibiotics based on the presented results from surface measurements of antibiotics in hospital wards in Sweden.

### Limitation of the study

One limitation with this study is that it was performed as an observational study with one measurement per ward. We therefore lack information of how levels vary over time and thus cannot make any conclusion if the contaminations detected in the study remains overtime or if they originate from current spills. Another limitation is that the information that was gathered from the wards concerning which drugs that were handled did not fully cover the form of the drug and thus we could not know if the detected antibiotics come from spills from particulate compounding or if it originates from handling of tablets or solutions for peroral administration making it more difficult to trace the source of the contamination. Moreover, in this study, wards were selected based on use of high amounts of antibiotics. A limitation is therefore that no measurements were carried out at wards where less or no amount of antibiotics were handled, which could be interesting to evaluate patterns of contamination. Also, for some sampling locations (surface categories), only a few samples were collected, which means that the result is more uncertain for these cases. Specifically, for the detection of antibiotics on the outer surface of a drug vial of CLOXA, further studies would be warranted to conclude if it is a major source of contamination. It has previously been shown, eg for antineoplastic drugs, that outer surfaces of primary drug vials can be contaminated with the drug it contains (Hilliquin et al. 2020; Cotteret et al. 2022; Kåredal et al. 2024).

## Conclusion

The developed wipe test method is user-friendly and can be used to determine low levels of surface contaminations of several of the most frequently used antibiotics in Swedish healthcare. Several of the antibiotics were detected in a high frequency of samples, and contamination of antibiotics was not only detected in medicine rooms but also in utility room, patient rooms, and common areas such as corridors indicating a widespread environmental contamination. In the medicine rooms, there were however significantly lower levels when closed systems were used supporting earlier findings that this safety measure helps to reduce contamination. The results also suggest that current cleaning routines do not sufficiently reduce spills and leakage regardless of source. Furthermore, the positive detection of antibiotics in many different samples from different locations suggests that spills and leakage likely arise not only from the compounding but also from handling and administration of drug tablets, antibiotic contaminated waste as well as other sources such as patients themselves and contaminated drug vials. Finally, the results in this study indicate that healthcare workers potentially can be dermally exposed to antibiotics due to high surface contaminations in hospital wards.

## Supplementary Material

wxaf067_Supplementary_Data

## Data Availability

The data underlying this article will be shared on reasonable request to the corresponding author.
