# Supplementary material for: Development and application of a wipe sampling method for detection of antibiotic surface contamination in hospital wards
Source: Ann Work Expo Health. 2025 Oct 23;70(1):wxaf067. doi: 10.1093/annweh/wxaf067 (PMC12821367; doi:10.1093/annweh/wxaf067)
Supplement: wxaf067_Supplementary_Data [file wxaf067_supplementary_data.pdf]

Supplementary information

Development and application of a wipe sampling method for detection of antibiotic surface contamination in hospital wards

Carina A. Nilsson[1], Elizabeth Huynh[1], Dallal Rashdan[1], Andreas Tinnert[1], Maria Hedmer[1,2], Monica Kåredal[1,2]

[1]Department of Occupational and Environmental Medicine, Skåne University Hospital, SE-22381 Lund, Sweden
[2]Division of Occupational and Environmental Medicine, Department of Laboratory Medicine, Lund University, SE-22100 Lund, Sweden

LC-MS/MS method

An LC-MS/MS method based on multiple reaction monitoring (MRM) assays were developed for the simultaneous detection of six antibiotics and their corresponding isotope-labelled antibiotics included as internal standard (IS), in total 12 compounds, Table S1. Quadrupole (Q)1 and Q3 m/z fragments of the MRMs were selected and the MS-parameters optimized for each antibiotic by flow injection analysis using acidified solutions of each compound (100 ng/mL in 0.5% acetic acid).

Separation was obtained in a 6-minute run with a flowrate of 0.4 mL/min in a gradient elution of mobile phase A (0.5% acetic acid) and B (0.5% acetic acid in methanol); isocratic at A:B 85:15 for 0.7 min, then to A:B, 40:60 for 1.3 min, to A:B 10:90 for 1 min, isocratic at A:B 10:90 for 1.5 min and then back to A:B 85:15 for 0.5 min where it remained for 1 min.

Table S1. Multiple reaction monitoring (MRM)s of each m/z included in MS-method operated in the positive ion mode. Ciprofloxacin (CIPRO), Cloxacillin (CLOXA), Piperacillin (PIPER), Penicillin V (PENV), Cefotaxime (CEFO) and Metronidazole (METRO).

**Antibiotic**					**IS[a]**		**t_R[b] (min)**
**Name**	**Fragment type**	**Q1[c]/Q3**	**DP[d]**	**CE[e]**	**Name**	**Q1/Q3**	
METRO-1	analyte	172.2/128	50	20	METRO-IS-1	176.2/128.2	0.74
METRO-2	control	172/82	50	34	METRO-IS-2	176.2/82.1	
CIPRO-1	analyte	332.2/314	90	31	CIPRO-IS-1	340/322	2.0
CIPRO-2	control	332/231	90	50	CIPRO-IS-2	340/235	
PENV-1	analyte	351/160.1	60	20	PENV-IS-1	356.2/160.1	2.7
PENV-2	control	351/114	60	48	PENV-IS-3	356.2/114.2	
CLOXA-1	analyte	436.1/277.1	50	20	CLOXA-IS-1	440/281	2.8
CLOXA-2	control	436.1/160.2	50	18	CLOXA-IS-2	440/160	
CEFO-1	analyte	456.1/396.2	70	16	CEFO-IS-1	459.2/399.2	1.7
CEFO-2	control	456.1/324	70	21	CEFO-IS-2	459.2/327	
PIPER-1	analyte	518.2/143.2	40	30	PIPER-IS-1	523/148	2.5
PIPER-2	control	518.2/359	40	15	PIPER-IS-2	523/364	

[a]IS = internal standard,
[b]t_R = retention time
[c]Q = Quadrupole
[d]DP = declustering potential
[e]CE = collision energy

Table S2. Screening campaign, characteristics of monitored workplaces where antibiotics were handled.

Hospital	Ward	Preparation of AB	Technical control measures	Personal protective equipment	Cleaning routines
A	Infectious disease unit	Medicine room	Safety cabinet, spike[b], infusion set	Protective gloves[c]	Workbench cleaned with surface disinfectant once per workday, work area in safety cabinet a couple of times per week
A	Intensive care unit	Medicine room	Spike	Protective gloves, disposal apron, respiratory protection	Workbench cleaned with surface disinfectant at the end of workday
A	Pediatric medicine	Medicine room	CSTD[d], spike, disposal absorbent mat on workbench	Protective gloves	Workbench cleaned with surface disinfectant before and after preparation
B	Surgery	Medicine room	CSTD, spike, disposal absorbent mat on workbench	Protective gloves	Workbench cleaned with surface disinfectant before and after preparation
B	Medicine	Medicine room	CSTD, spike, disposal absorbent mat on workbench	Protective gloves	Workbench cleaned with surface disinfectant before and after preparation
B	Infectious disease unit	Medicine room	Forced general ventilation, spike, disposal absorbent mat on workbench	Protective gloves and coat	Workbench cleaned with surface disinfectant before and after preparation
B	Orthopedics	Operating room	Infusion set, disposal absorbent mat	Protective gloves and coat, disposal apron, face mask, surgical hood	Workbench cleaned with surface disinfectant after preparation
C	Medicine	Medicine room	Ventilated perfusion bench, CSTD, spike	Protective gloves, apron, face mask	Workbench cleaned with surface disinfectant after preparation
C	Orthopedics	Medicine room	CSTD, spike, disposal absorbent mat on workbench	Protective gloves	Workbench cleaned with surface disinfectant and water every morning
C	Surgery	Medicine room	Ventilated perfusion bench, CSTD, spike	Protective gloves	Workbench cleaned with surface disinfectant every morning and after spill
D	Medicine	Medicine room	CSTD, compounding carried out on tray of stainless steel	Protective gloves	Workbench cleaned with disinfectant after preparation. Floors cleaned once per day, storage shelves once per month.
D	Infectious disease unit	Medicine room	CSTD or spike, disposal absorbent mat on workbench	Protective gloves	Workbench cleaned with surface disinfectant once per day. New disposal absorbent mat.
D	Orthopedics	Medicine room	CSTD, disposal absorbent mat on workbench	Protective gloves	Workbench cleaned with surface disinfectant once per day.
E	Medicine	Medicine room	CSTD, disposal absorbent mat on workbench	Protective gloves, disposal apron	Workbench cleaned with surface disinfectant after preparation
E	Surgery	Medicine room	CSTD or spike, disposal absorbent mat on workbench	Protective gloves	Workbench cleaned with surface disinfectant after preparation
E	Infectious disease unit	Medicine room	Safety cabinet, CSTD, spike, disposal absorbent mat on workbench	Protective gloves	Workbench cleaned with surface disinfectant after preparation

[a]AB = antibiotics, [b]Air vented dispensing units with an inbuilt particle filter (open system), [c]Commonly made of nitrile, [d]CSTD = closed system drug-transfer device

Table S3. List of question in questionnaire

**Question**
Which antibiotics that were handled?
Was a risk assessment regarding handling of antibiotics performed?
Frequency of antibiotic preparations?
Who performs the preparations and where are they made?
Which technical safety equipment is used during preparations?
Which personal protective equipment is used?
How is the antibiotics-contaminated waste disposed?
Describe the cleaning routine after preparation of antibiotics.
How often is cleaning performed?
Who is cleaning?

Matrix effects

Matrix effects due to wipe material were evaluated by assessing the signal of individual MRMs in calibration samples with known amounts of antibiotics and with and without wipe tissue which were then treated according to the sample work-up procedure. The difference between the slope of the calibration curve based on the peak area of each analyte in standards prepared and analyzed with tissue added and in samples without tissue added was compared: CIPRO 100%, CLOXA 46%, PIPER 70%, PENV 58%, CEFO 26% and METRO 12%, Table S4. When the calibration curve instead was based on peak area ratios between analyte and the corresponding IS the differences were: CIPRO 12%, CLOXA 21%, PIPER 16%, PENV 22%, CEFO 1.7% and METRO 8.0% thus showing that matrix effects indeed occur due to the presence of tissue but that a major part of the effects are reduced by using internal standards for quantification. Thus, tissue was used in all standards prepared for quantification.

Table S4. Difference in the slope of a standard curve constructed from standards with and without addition of matrix (i.e. wipe tissues, containing 0.1-200 ng/sample) assessed by A) using the analyte peak area or B) using peak area ration between the analyte and the corresponding internal standard (IS).

	Antibiotics					
Difference in slope based on	CIPRO	CLOXA	PIPER	PENV	CEFO	METRO
(A) Peak area (%)	100	46	70	58	26	12
(B) Peak area ratio analyte/IS (%)	12	21	16	22	1.7	8.0

Precision and accuracy

Within-day and between-day precisions were determined at a higher (QC_{High}) and lower quality control samples (QC_{Low}) and results are presented in Table S5 and S6.

A defined area of 400 cm^2 was spiked with a mixture with known amounts of each antibiotic (100 ng per compound, 100 µL of a solution containing all drugs with concentration 1000 ng per substance/mL in ultrapure water). Wipe sampling and sample work-up and analysis was performed according to the protocol. Defined areas composed of three different surface materials were spiked (*n* = 10 per surface material); stainless steel, laminate and plastic. Surface recovery was calculated as the percentage (ratio) between the concentration of spiked and wiped surface samples treated according to the sample work-up procedure and the concentration of a quality control sample (containing wise tissue) spiked with the same amount of antibiotics (subjected to the sample work-up procedure) and the results are presented in Table S7.

Table S5. Within-day precision and accuracy determined at a high and low level in samples spiked with a mixture of antibiotics that then were subjected to the sample work-up procedure.

	CIPRO	CLOXA	PIPER	PENV	CEFO	METRO
			QC_{High} 100 ng/sample			
mean[a]	78	89	106	75	104	99
accuracy (%)	78	89	106	75	104	99
RSD[b] (%)	18	2.5	3.7	3.0	2.7	2.5
			QC_{Low} 5 ng/sample			
mean[a]	4.1	4.4	4.9	3.9	5.2	5.5
accuracy (%)	82	88	98	77	104	110
RSD[b] (%)	22	4.7	4.8	11	4.1	4.8

[a] Spiked samples (QC, $n = 10$)
[b] RSD = relative standard deviation

Table S6. Between-day precision and accuracy determined at a high and low level in samples spiked with a mixture of antibiotics at 5 different occasions over three weeks that then were subjected to the sample work-up procedure.

	CIPRO	CLOXA	PIPER	PENV	CEFO	METRO
			QC_{High} 100 ng/sample			
mean[a]	98	85	105	73	102	99
accuracy (%)	98	85	105	73	102	99
RSD[b] (%)	26	6.9	7.6	5.7	3.5	7.6
			QC_{Low} 5 ng/sample			
mean[a]	5.6	4.3	5.1	3.6	5.0	5.2
accuracy (%)	113	86	103	72	100	104
RSD[b] (%)	21	8.9	3.7	6.2	6.7	7.2

[a] Spiked samples (QC, $n = 2$, at 5 occasions)
[b] RSD = relative standard deviation

Table S7. Recovery of antibiotic levels of wipe sampled surfaces (100 ng per surface, n = 10 per surface material). Recovery (%) assessed with quantification of levels from in wipe sampled surfaced compared to samples spiked with the same amount (QC 100 ng per sample).

	**Mean recovery (%)[a]±SD[b]**					
**Surface material[c]**	**CIPRO**	**CLOXA**	**PIPER**	**PenV**	**CEFO**	**METRO**
Stainless steel	16±8.0	44±19	55±13	37±20	64±8.7	93±11
PVC	59±36	71±16	72±16	45±16	92±21	106±12
Laminate[d]	57±28	69±14	77±10	65±9.7	82±10	88±6.9

[a] Surface recovery was determined from the ratio between the obtained concentration of sample from spiked surfaces that were wipe sampled and treated according to the work-up procedure and the obtained concentration of a spiked sample containing wipe tissue and then subjected to the work-up procedure. $n = 10$ for each surface material and method.
[b] SD = standard deviation
[c] Three surface materials were tested: (A) stainless steel, plastic material, and laminate, by spiking them with 100 ng per antibiotic.
[d] One result excluded, outlier

Stability

The stability of each antibiotics stored at room temperature, at 4 °C, and at -20 °C was assessed by spiking wipe samples (two tissues in 50-mL Falcon tubes, 100 ng/compound (100 µl of a mixture containing all antibiotics C=1000 ng/mL in ultrapure water)) and then determining the remaining level at time-points 0, 1, 2, 7, 14 days, 1, 2 and 6 months (n = 5 per time-point and temperature), see Table S8.

Table S8. Recovery of Ciprofloxacin, Cloxacillin, Piperacillin, Penicillin V, Cefotaxime and Metronidazole in spiked samples (*n* = 5 per analysis time and temperature) stored at room temperature, at 4 °C and -20 °C.

		**Room temperature**			**4 °C**			**-20 °C**		
**Antibiotics**	**Time (days)**	**Amount (ng/sample)**	**Recovery (%)**	**RSD[a] (%)**	**Amount (ng/sample)**	**Recovery (%)**	**RSD (%)**	**Amount (ng/sample)**	**Recovery (%)**	**RSD (%)**
CIPRO	0	92	100	24	92	100	31	92	100	31
CIPRO	1	98	107	3.3	101	110	11	101	110	6.0
CIPRO	2	125	136	6.4	120	131	10	141	154	5.1
CIPRO	7	133	145	7.5	152	165	14	177	193	13
CIPRO	14	101	111	17	117	128	10	125	137	12
CIPRO	28	130	142	11	148	161	14	189	206	7.1
CIPRO	56	106	116	38	97	106	7.3	123	134	28
CIPRO	196	NA[b]	-	-	107	117	11	140	153	3.8
CLOXA	0	85	100	8.7	85	100	8.7	85	100	8.7
CLOXA	1	83	97	6.1	90	105	5.0	94	110	4.4
CLOXA	2	64	75	13	76	89	2.4	83	98	3.5
CLOXA	7	49	57	5.8	57	66	13	79	93	3.9
CLOXA	14	30	35	6.7	29	34	33	77	91	2.4
CLOXA	28	16	18	23	ND[c]	-	-	84	98	4.5
CLOXA	56	1.1	1.3	33	ND	-	-	68	80	5.2
CLOXA	196	NA	-	-	ND	-	-	24	28	15
PIPER	0	101	100	9.9	101	100	9.9	101	100	9.9
PIPER	1	102	100	4.8	104	103	3.2	109	108	3.4
PIPER	2	92	91	7.4	101	100	2.8	108	106	2.5
PIPER	7	76	75	4.3	90	89	11	106	105	5.8
PIPER	14	51	50	6.3	59	58	30	105	104	1.8
PIPER	28	25	25	10	1.9	1.9	146	112	111	5.1
PIPER	56	6.1	6.1	13	ND	-	-	95	94	6.0
PIPER	196	NA	-	-	ND	-	-	50	49	10
PenV	0	73	100	7.8	73	100	7.8	73	100	7.8
PenV	1	74	102	3.1	77	105	4.9	80	109	2.1
PenV	2	64	88	7.2	66	90	1.1	72	99	2.0
PenV	7	54	75	3.1	62	85	11.7	73	100	4.6
PenV	14	40	54	4.2	40	55	24	69	95	4.1
PenV	28	24	33	9.7	1.9	2.6	159	71	98	5.6
PenV	56	6.6	9.1	5.8	ND	-	-	62	85	2.8
PenV	196	NA	-	-	ND	-	-	34	47	12
CEFO	0	97	100	7.6	97	100	7.6	97	100	7.6
CEFO	1	102	105	2.7	106	109	1.8	105	109	2.6
CEFO	2	101	104	3.0	103	106	1.9	109	112	0.9
CEFO	7	77	79	5.2	95	98	7.4	104	107	3.0
CEFO	14	57	58	4.8	88	91	14	108	111	3.1
CEFO	28	30	31	4.1	33	34	36	111	115	4.0
CEFO	56	8.0	8.3	4.1	4.3	4.4	93	98	101	2.7
CEFO	196	NA	-	-	ND	-	-	96	99	3.9
METRO	0	90	100	8.7	90	100	8.7	90	100	8.7
METRO	1	103	115	5.3	101	112	2.9	101	112	4.9
METRO	2	117	131	2.8	109	122	4.8	113	126	3.3
METRO	7	101	112	2.1	100	112	6.6	104	116	3.9
METRO	14	108	120	4.2	107	119	5.3	109	121	4.5
METRO	28	103	115	1.6	99	110	10	101	112	1.7
METRO	56	112	124	2.2	111	123	5.6	111	123	2.7
METRO	196	NA	-	-	79	88	2.6	86	95	3.0

[a] RSD = relative standard deviation

[b] NA = not analyzed

[c] ND = not detected (below the LOQ)

Table S9. Environmental surface concentration of antibiotics determined at the hospital wards shown as median, ranges and the percentage of samples detected above the LOQ.

Hospital	Ward	n[a]	CIPRO		CLOXA		PIPER		PENV		CEFO		METRO	
			**Median (min-max) (pg/cm^2)**	**>LOQ[b] (%)**	**Median (min-max) (pg/cm^2)**	**>LOQ (%)**	**Median (min-max) (pg/cm^2)**	**>LOQ (%)**	**Median (min-max) (pg/cm^2)**	**>LOQ (%)**	**Median (min-max) (pg/cm^2)**	**>LOQ (%)**	**Median (min-max) (pg/cm^2)**	**>LOQ (%)**
A	Infectious disease unit	22	140 (ND[c]-9600)	68	340 (ND-1.6×10^6)	86	4900 (48-7.6×10^5)	100	ND (ND-65)	14	4000 (55-27×10^6)	100	46 (ND-420)	95
A	Intensive care unit	20	ND (ND-40)	5	21 (ND-2500)	75	110 (ND-1.4×10^4)	75	ND (ND-ND)	0	190 (ND-1.0×10^6)	90	ND (ND-330)	35
A	Pediatric medicine	31	ND (ND-ND)	0	ND (ND-120)	3	78 (ND-1.7×10^6)	74	8 (ND-1.0×10^6)	52	73 (ND-6.5×10^5)	74	8 (ND-430)	61
B	Surgery	18	ND (ND-82)	33	16 (ND-2.8×10^5)	72	160 (ND-3100)	83	ND (ND-ND)	0	69 (ND-2.7×10^4)	72	95 (ND-5200)	78
B	Medicine	20	ND (ND-3.8×10^5)	40	210 (ND-2.5×10^4)	80	ND (ND-1300)	45	ND (ND-2.6×10^5)	15	150 (ND-6200)	95	ND (ND-1100)	30
B	Infectious disease unit	19	ND (ND-ND)	0	2300 (ND-1.8×10^5)	95	330 (ND-1.3×10^5)	95	ND (ND-ND)	0	51 (3.0-4000)	100	67 (ND-3100)	84
B	Orthopedics	5	ND (ND-ND)	0	48 (5.9-380)	100	91 (ND-2200)	80	ND (ND-ND)	0	ND (ND-57)	20	ND (ND-9,4)	40
C	Medicine	19	ND (ND-1200)	11	84 (ND-3.1×10^4)	84	14 (ND-5.8×10^5)	74	ND (ND-140)	11	310 (ND-1.0×10^5)	95	14 (ND-2500)	79
C	Orthopedics	16	ND (ND-77)	13	64 (ND-4.2×10^4)	56	360 (ND-6.6×10^5)	81	ND (ND-ND)	0	1200 (36-5.9×10^5)	100	ND (ND-85)	31
C	Surgery	25	ND (ND-1.9×10^4)	48	ND (ND-72)	16	300 (ND-1.3×10^5)	68	ND (ND-ND)	0	16 (ND-3.8×10^5)	80	65 (ND-1900)	84
D	Medicine	16	ND (ND-ND)	0	110 (ND-1800)	81	870 (ND-1.1×10^5)	94	ND (ND-ND)	0	660 (9.8-3.7×10^5)	100	1.6 (ND-180)	50
D	Infectious disease unit	21	ND (ND-75)	10	100 (ND-2200)	81	90 (ND-4300)	81	ND (ND-38)	29	62 (ND-9100)	76	ND (ND-790)	29
D	Orthopedics	19	ND (ND-58)	21	870 (11-1.8×10^6)	100	110 (ND-2.5×10^4)	89	ND (ND-52)	21	190 (ND-2.6×10^4)	84	ND (ND-280)	47
E	Medicine	19	ND (ND-540)	5	ND (ND-1.2×10^4)	47	400 (ND-3.0×10^6)	89	ND (ND-ND)	0	48 (ND-3.1×10^4)	68	ND (ND-75)	16
E	Surgery	21	ND (ND-ND)	0	4.6 (ND-5700)	52	620 (ND-9.3×10^4)	90	ND (ND-ND)	0	1000 (ND-4.6×10^6)	95	ND (ND-1400)	38
E	Infectious disease unit	22	ND (ND-1.4×10^5)	41	53 (ND-9900)	73	130 (ND-9.4×10^5)	77	6.2 (ND-2.0×10^4)	50	13 (ND-4.8×10^4)	64	ND (ND-410)	27

[a]Number of wipe tests analyzed

[b]Percentage of samples above the limit of quantification (LOQ)

[c]ND = not determined, value below the limit of quantification
